# Persistent RNA SARS-CoV-2 Detection in a HIV-Infected Patient

**DOI:** 10.3390/healthcare10060982

**Published:** 2022-05-25

**Authors:** Lucian Giubelan, Ilona Stanciu, Cristina Ilie, Vlad Pădureanu

**Affiliations:** 1 Department of Infectious Diseases, University of Medicine and Pharmacy of Craiova, 200349 Craiova, Romania; ligiubelan@yahoo.com (L.G.); vldpadureanu@yahoo.com (V.P.); 2 Department of Infectious Diseases, Victor Babes Hospital of Infectious Diseases and Pulmonology, 200515 Craiova, Romania; 3 Department of Internal Medicine, University of Medicine and Pharmacy of Craiova, 200349 Craiova, Romania

**Keywords:** RNA SARS-CoV-2, HIV, polymerase chain reaction

## Abstract

The aim of this paper is to present a case of COVID-19 in a newly diagnosed HIV-infected, severely immunodepressed patient with a long persistence of positive RT-PCR for RNA SARS-CoV-2. Indirect data suggests that viable virus persisted for a long time in the absence of an adequate defense of the host. Improved immunity after starting antiretroviral treatment was not associated with an increased inflammatory reaction as regarding the infection due to the coronavirus and, apparently, was the main factor to control the infection. Remdesivir used to combat the SARS-CoV-2 infection had no immediate effect on the recorded cycle-threshold.

## 1. Introduction

Reverse transcriptase polymerase chain reaction (RT-PCR) to detect ribonucleic acid (RNA) of Severe Acute Respiratory Syndrome Coronavirus 2 (SARS-CoV-2) is the most accurate diagnosis test in clinical practice, while viral culture remains reserved for well-equipped research laboratories. In an infected individual, the time for viral clearance differs based on the involved test. The median time of viral persistence is 7 days for virus culture and 34 days based on RNA detection in nasopharyngeal and oropharyngeal samples [1], however another study found that the mean lower bound of viral RNA persistence is 17.3 days, while the mean upper limit goes to 22.7 days [2]. Correlations between SARS-CoV-2 RNA detection and viable virus presence are based on the cycle-threshold for RT-PCR testing, an average value of less than 28.4 being the limit for virus isolation (with variations between 24 and 37) [1,3]. There are reports of much longer RNA viral shedding (including recurrences of viremia) in respiratory samples (from 60 to 268 days) in some particular cases, mainly in immunocompromised patients [4,5,6]. Factors for prolonged RNA SARS-CoV-2 persistence include older age, female gender, an interval of more than 10 days from symptom onset and hospital admission, or immunodepression [4,7]. Clinical and epidemiological significance of persistent RNA SARS-CoV-2 remains unclear, and the best practical approach for such patients is yet to be determined.

The aim of this study is to present a case of long persistence of the SARS-CoV-2 RNA in a newly diagnosed HIV-infected patient (from the Clinical Case Records of “Victor Babes” Hospital, Craiova, Romania).

## 2. Case Report

The patient P.C. is a male born in 1967, living an urban area. He was in close contact with another person infected with SARS-CoV-2, got tested on 30 March 2021, and was found positive. He had a mild form of Coronavirus infectious diseases 2019 (COVID-19) and he chose to be treated at home with symptomatic drugs. In June 2021 he was admitted in an Internal Medicine department to be treated for a persistent anemia and he was also found positive for rapid SARS-CoV-2 antigen testing on June 5 and 15. The RT-PCR testing was performed in June 16, the result was positive, but it was considered as a post-COVID-19 status. A month later the patient repeated an antigenic test, the result was positive, and he decided to consult an infectious disease specialist. When admitted in our department (on July 18) he was alert, apparently in well condition, but pale, with oral thrush, mild liver enlargement, and a slightly raised red–purple lesion of about 5 cm in diameter located on his left sole. The height of the patient was 179 cm and the weight was 62 kg (body-mass index—BMI—19.4). His blood pressure was normal, he was tachycardic (115 beats per minute), his respiratory rate was 20 per minute, and the oxygen saturation was 96% while breathing room air. He was also complaining of losing weight (about 20 kilos in the last 4 months). Epidemiologically, he stated that he was not vaccinated against SARS-CoV-2, he did not travel abroad in the past 6 months, and he admitted he had multiple sexual contacts in the past without using protective measures. His initial laboratory tests showed: hemoglobin level 8.8 g/dL, leukocytes count 3100/mm^3^, 43% lymphocytes and 9% monocytes in the white blood formula, platelets count 246,000/ mm^3^, eritrocytes sedimentation rate (ESR) 95 mm/hour, D-dimers 514.65 ng/mL (normal range: 0–500), C reactive protein 12.5 μg/mL (normal range: 0–10); the rest of the data were within normal limits. A computed-tomography (CT) scan of the lungs showed diffuse ground-glass opacities in the superior parts of both lungs, mainly subpleural localized. Brain CT was normal and abdominal ultrasound showed moderate liver enlargement. Dermatologic consultation raised the suspicion of Kaposi sarcoma and, later, based on a bioptic specimen sent for analysis, the diagnosis was confirmed.

Sputum sample was negative for *Mycobacterium tuberculosis* (GeneXpert MTB/RIF) An oral swab came positive for *Candida albicans* and the ELISA test for HIV also proved positive. On July 20 a new RT-PCR assay for SARS-CoV-2 detection resulted positive, with a cycle-threshold of 7.17 (see Supplementary Materials).

**Figure 1:**
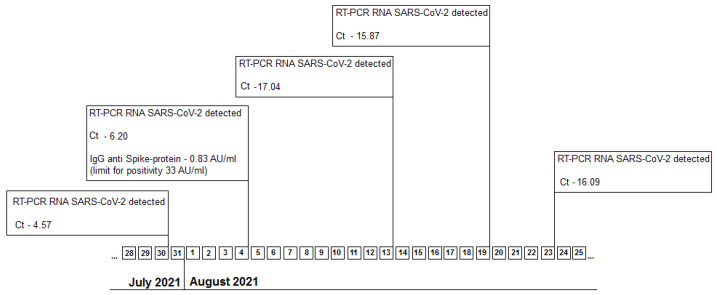
Timeline for real time RT-PCR testing for SARS-CoV-2 between 30 July and 23 August 2021. Legend: Ct-cycle-threshold.

Oropharyngeal swab sample was sent for genetic testing at the Regional Center for Medical Genetics from Craiova and later results came positive for the B 1.1.7 strain, showing the E484K and N501Y mutations (TaqMan^TM^ SARS-CoV-2 Mutation Panel). Later, on July 27, the viral load for HIV (HIV-VL) showed 930,000 viral copies/mL and the CD_4_ count was 25 cells/mL. Further real time RT-PCR testing for SARS-CoV-2 is reported in Figure 1.

During hospitalization he experienced asthenia during the first week, but otherwise his medical status was good. The laboratory tests showed leukopenia (1300/mm^3^ in August 16 and 1900/ mm^3^ in August 25). Additionally, his C reactive protein level increased three times above the normal value (30.91 μg/mL in August 16), only to drop to 13.93 μg/mL on the day of hospital release.

The patient was isolated in our department between 18 July and 23 August 2021. He started antiviral-oriented treatment in July 23 with Remdesivir, 200 mg i.v. initially, then 100 mg for another four days; he also received Enoxaparine 4000 i.u. (prophylactic dosage) and Ibuprofen 200 mg twice daily. Fluconazole 200 mg/day orally for ten days was also added to treat the oral candidiasis. On August 17, antiretroviral treatment was started with the combination Doravirine + Lamivudine + Tenofovir disoproxil (Delstrigo) 100/300/245 mg, orally, once daily.

The patient demanded to be released from the hospital on August 26. He was then referred and monitored (as an outpatient) by the HIV Regional Center from Craiova, Romania; he was asked to wear facial mask until the RT-PCR for SARS-CoV-2 result came negative and to get vaccinated against COVID-19. He also had continued the antiretroviral treatment.

On October 5 he was consulted in our department. He was in good clinical condition, but the Kaposi sarcoma worsened, with new lesions having emerged on his left calf and thigh and also on the right side of the abdomen, thought to be an immune reconstitution inflammatory syndrome however a new CD_4_ count was not available. The RT-PCR assay for SARS-CoV-2 detection resulted negative and the IgG anti Spike-protein level was 51 AU/mL (positive); later came the result for the HIV-VL (236 copies/mL). He was sent to an oncology department, tested negative for SARS-CoV-2 (rapid antigen test), and underwent local surgery to partially remove the lesions; he also started chemotherapy in December 2021 in order to remit the Kaposi sarcoma. The first option was for liposomal Doxorubicine, but the patient developed an allergic reaction to the formulation, and it was switched to Paclitaxel 200 mg every 2 weeks.

At the end of January 2022 he was in good clinical condition, gained 10 kg in weight, and there was only a remnant lesion of the Kaposi sarcoma on his left sole; he continued the antiretroviral treatment and chemotherapy. In March 2022 the HIV-VL was under the detection limit (40 copies/mL) and the CD_4_ count was 145 cells/mm^3^.

## 3. Discussion

This is the longest persistence of RNA SARS-CoV-2 (147 days from March 30 until August 23) recorded in our clinic and one of the longest in medical literature, according to the authors’ knowledge. Starting from the first positive RT-PCR until the negative test it was 190 days, but it is not certain when exactly the virus disappeared, since there was no testing between the release from the infectious diseases department and the consultation in October 5.

The dominant viral variant in Romania during March 2021 was B 1.1.7 (the third wave of the pandemic), while the earliest detection of the delta variant, B.1.617.2, was at the end of August 2021. In our case we have detected the alpha variant of the SARS-CoV-2 in July 2021. We do not have definitive proof that this was the initial SARS-CoV-2 variant, but, taking into account the epidemiological data, the series of positive RT-PCR tests and the fact that reinfections are rare in a time period of 90 days from the initial diagnosis [8,9], this hypothesis is most probable.

Escape mutations were detected in other immunocompromised patients diagnosed with COVID-19. It seems that the chances of occurring and the number of mutations are linked with the period of time in which the virus replicates itself, but, also, treatment with certain monoclonal antibodies against SARS-CoV-2 may force the microorganism to mutate [10,11]. Unfortunately, for the presented case it was not possible to perform a phylogenetic analysis.

Remdesivir is used to treat COVID-19 patients, as well as other infections due to beta-coronaviruses [12], and its benefits seems to be linked to the early use of the drug [13]. For our patient we have assumed that, due to the immunodepression, there was a continuous viral replication (suggested by the RT-PCR cycle-threshold of 7.17) and therefore it was decided to use this medication. Three days after the treatment was stopped the value of the cycle-threshold remained low (4.57), still suggesting a high level of coronavirus replication, but two weeks later the value increased almost four times. Two studies have shown that Remdesivir was effective in their case, lowering the viral load, however being unable to clear the virus from the upper respiratory tract [5,6]. For the present case, it is not possible to evaluate the efficacy of antiviral treatment.

Baang and col. [14] have described a prolonged case of SARS-CoV-2 infection in an immunocompromised host (lymphoma and associated B-cell immunodeficiency) without seroconversion. Resolution of the case was found after 119 days after repeated treatments with Remdesivir and convalescent plasma (which may have provided the necessary antibodies to fight the virus). Niyokuru and col. [15] also presented two cases of immunocompromised patients (recipients of liver and bone marrow transplants) infected with SARS-CoV-2, for which there is evidence of attenuated humoral immunity. The cases pointed out the importance of the humoral immunity in ending SARS-CoV-2 infection. In our case it was proved that seroconversion occurred after controlling the HIV infection, and it was associated with the ending of PCR positivity.

After the start of the antiretroviral treatment, concerns have been raised that the COVID-19 may evolve to a severe form, due to improved immunity and, consequently, the inflammatory response. However, the clinical evolution and the lab tests pointed to a mild form until the RT-PCR assay became negative.

As seen in other complex medical cases, a multidisciplinary approach is an important factor for improving survivability [16]. For the present case, cooperation between specialists (infectious diseases, genetics, internal medicine, dermatology, and oncology) was the key factor for successful management of the patient.

## 4. Conclusions

This case shows the persistence of COVID-19 in a HIV-infected, severely immunodepressed patient. Indirect data suggests that viable virus persisted for a long time in the absence of an adequate defense of the host. Improved immunity after starting antiretroviral treatment was not associated with an increased inflammatory reaction as regarding the infection due to the coronavirus and, apparently, was the main factor to control the infection. Remdesivir used to combat the SARS-CoV-2 infection had no immediate effect on recording the cycle-threshold.

## Supplementary Materials

The following are available online at https://www.mdpi.com/article/10.3390/healthcare10060982/s1, Figure S1: Patient P.C., first cycle thresold determined for the case, suggesting active viral replication; red line—detected sample, blue line—comparator; Abbott m2000rt machine.

## Abbreviations

AU	arbitrary units
BMI	body mass index
CD_4_	cluster of differentiation 4
COVID-19	Corornavirus infectious disease 2019
CT	computed-tomography (imagery)
cm	centimeter(s)
ELISA	enzyme-linked immunosorbent assay
ESR	erytrocytes sedimentation rate
g/dL	grams/deciliter
HIV	human immunodeficiency virus
HIV-VL	HIV viral load
i.u.	international units
i.v.	intravenous route
kg	kilogram(s)
mg	milligram(s)
μg	microgram(s)
ng	nanogram(s)
mL	milliliter(s)
mm^3^	cubic millimeter(s)
RNA	ribonucleic acid
RT-PCR	Reverse transcriptase polymerase chain reaction
SARS-CoV-2	Severe Acute Respiratory Syndrome Coronavirus 2
