# Supplementary material for: Persistent RNA SARS-CoV-2 Detection in a HIV-Infected Patient
Source: Healthcare (Basel). 2022 May 25;10(6):982. doi: 10.3390/healthcare10060982 (PMC9222592; doi:10.3390/healthcare10060982)
Supplement: Supplementary file 1 [file healthcare-10-00982-s001.zip › healthcare-1708226-supplementary.pdf]

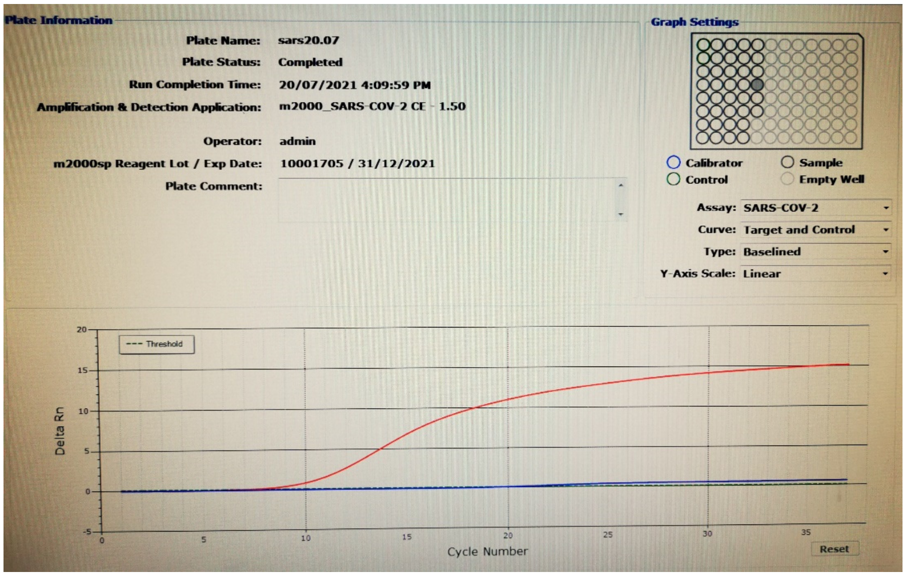

Figure S1. – Patient P.C., first cycle thresold determined for the case, suggesting active viral replication; red line – detected sample, blue line – comparator; Abbott m2000rt machine.
